# Comparative evaluation of fiber and metal posts on mandibular bone microstructure: a fractal dimension analysis

**DOI:** 10.1186/s12903-026-09341-z

**Published:** 2026-07-17

**Authors:** Burcu Kızılırmak, Hatice Yılmaz, İbrahim Burak Yüksel, Neslihan Güntekin

**Affiliations:** 1 Department of Prosthodontics, Faculty of Dentistry, Çanakkale Onsekiz Mart University, Çanakkale, 17110 Türkiye; 2 Department of Oral and Maxillofacial Radiology, Faculty of Dentistry, Necmettin Erbakan University, Konya, 42090 Türkiye; 3 Department of Prosthodontics, Faculty of Dentistry, Necmettin Erbakan University, Konya, 42090 Türkiye

**Keywords:** Fiber post, Fractal analysis, Metal post, Panoramic radiography, Trabecular bone complexity

## Abstract

**Background:**

The aim of this study was to quantitatively evaluate the effects of prefabricated fiber and metal posts on mandibular trabecular bone microarchitecture by comparing fractal dimension values derived from panoramic radiographs at pre-treatment and post-treatment time points.

**Methods:**

A cohort of 101 individuals (50 fiber posts, 51 metal posts) was analyzed. To ensure comparability, both groups were matched regarding age, gender (p > 0.05). Digital panoramic radiographs were obtained at pre-treatment and post-treatment time points. Images were saved in uncompressed Tagged Image File Format and processed using ImageJ software. Bone complexity was quantified using the White and Rudolph box-counting method. Standardized Regions of Interest (50 × 50 pixels) were precisely defined in the peri-radicular bone, strictly excluding anatomical landmarks such as the mandibular canal and mental foramen. Intra-observer reliability was verified via the Intraclass Correlation Coefficient. Due to the non-normal distribution (Shapiro-Wilk test), data were analyzed using Mann–Whitney U and Wilcoxon signed-rank tests.

**Results:**

There was no statistically significant difference between pre- and post-treatment fractal dimension values within the fiber and metal post groups (p > 0.05). Inter-group comparisons revealed no significant disparity in trabecular bone complexity between the two post systems (p > 0.05). Furthermore, gender did not exert a significant influence on the fractal metrics. Sensitivity power analysis confirmed that the sample size was sufficient to detect clinically relevant changes.

**Conclusion:**

The findings indicate that prefabricated fiber and metal posts produce similar short-term effects on mandibular trabecular bone microarchitecture, with no significant difference in fractal dimension values either within or between groups. From a clinical perspective, fractal analysis did not reveal a detectable difference in peri-radicular trabecular bone complexity between the two post systems over a one-year follow-up; this radiograph-based observation may provide an additional consideration for material selection when esthetic, financial considerations are weighed.

## Background

Post-core applications are fundamental in restoring the structural integrity and functionality of endodontically treated teeth with extensive coronal loss. In cases where remaining tooth structure is insufficient due to trauma, deep caries, or extensive periapical involvement, intraradicular posts are essential for providing adequate retention for the definitive restoration [1, 2]. Current clinical practice utilizes various post materials, including metal, ceramic, and fiber-reinforced polymers, each possessing distinct elastic moduli. These mechanical disparities significantly influence stress distribution within the radicular dentin and, consequently, the long-term clinical success of the restoration. However, a definitive consensus on the optimal post system remains elusive [3, 4]. Although traditional cast post-cores and prefabricated metal posts are widely used, catastrophic root fractures can occur. Glass or carbon fiber reinforced posts, owing to their elastic moduli similar to those of human dentin, have been widely advocated as a more biomechanically favorable alternative, with this dentin-like elasticity theoretically influencing stress distribution and reducing the risk of root fracture [4–7].

Post-core applications in endodontically treated teeth cannot fully provide natural stress distribution because materials with different mechanical properties than dentin are used, and this situation may increase the risk of biomechanical failure. The elastic modulus of the post material is decisive in this process, and glass fiber posts with values close to those of dentin offer more homogeneous stress distribution and a lower risk of root fracture [6, 7]. In contrast, metal posts with high rigidity can cause stress concentration in the dentin. With increasing aesthetic expectations, metal-free post systems with dentin-like properties have come to the fore. Although fiber posts are widely considered superior in terms of fracture resistance and biomechanical compatibility with dentin, whether this advantage extends to the biological response of the surrounding alveolar bone remains unclear. Whether these differences in mechanical properties have a significant effect on bone microarchitecture is of critical importance for tooth survival [8–10].

Crucially, the mechanical stress generated within the dentin is not isolated; it is transmitted to the surrounding alveolar bone, inducing remodeling within both the cortical and trabecular compartments. While finite element analysis (FEA) has provided theoretical insights into these load transfer mechanisms, clinical evidence directly evaluating the biological and structural responses of the alveolar bone to different post systems remains scarce [11]. Although the mechanical properties of the two post systems differ, the extent to which these differences affect the surrounding bone tissue has not yet been clearly established clinically. This gap in the literature highlights the necessity for objective methods to assess long-term bone architecture changes in response to intracanal restorations.

Fractal analysis (FA) has emerged as a robust, non-invasive mathematical tool for quantifying the complexity of irregular biological structures. By utilizing the White and Rudolph box-counting technique on digitized radiographs, FA provides a numerical value known as the fractal dimension (FD), which reflects the structural complexity and connectivity of the trabecular pattern [12–14]. Unlike traditional densitometry, FD is less sensitive to variations in radiographic exposure parameters and provides a more accurate characterization of bone microarchitecture. Higher FD values typically correlate with increased trabecular complexity, reflecting active angiogenesis and bone remodeling processes [15, 16]. FD, used in the assessment of alveolar bone demineralization, is a mathematical indicator that quantitatively defines the geometric texture and self-similarity properties of biological elements with a fractal structure. The naturally high porosity and hierarchical architecture of cancellous (spongy) bone make FD analysis extremely useful for comparing regional bone density and trabecular organization. Therefore, FA is a proven method that provides reliable results in evaluating trabecular bone structure in various dental pathologies, such as bone remodeling and maturation, and root canal treatment. Thanks to its non-invasive nature, scale independence, and minimal dependence on radiographic imaging parameters, FA has become an increasingly important diagnostic technique for monitoring bone health and architecture in dental images [13, 17, 18]. Although various FEA and in vitro studies have suggested that the effect of post-core materials on the surrounding bone may be limited, this assumption has not been validated longitudinally under clinical conditions using objective bone microarchitecture measures [5, 6]. This study presents the first longitudinal clinical evaluation to test such an effect using non-invasive fractal analysis. In this context, the present study aimed to evaluate and compare the longitudinal effects of prefabricated fiber and metal posts on the microstructure of mandibular trabecular bone using fractal analysis of panoramic radiographs under clinical conditions.

The research hypothesis predicted that fractal dimension values obtained from post-treatment panoramic radiographs would differ significantly between patients treated with prefabricated fiber posts and those treated with prefabricated metal posts.

## Methods

### Study design and ethical approval

This study was designed as a retrospective observational investigation to evaluate changes in mandibular bone microarchitecture following the placement of prefabricated fiber and metal posts. The study protocol strictly adhered to the ethical principles of the Declaration of Helsinki and received formal approval from the Institutional Review Board (IRB) and Ethics Committee of Necmettin Erbakan University (Decision No:2025/663). All data were accessed and processed in compliance with institutional privacy and data protection guidelines. The study population was retrospectively identified from the patient database of the Department of Prosthodontics, Faculty of Dentistry, Necmettin Erbakan University. The cohort consisted of individuals (aged 18–80 years) who had undergone post-core restoration. Data retrieval and radiographic assessments were conducted using the comprehensive medical and imaging archives maintained by the Department of Oral and Maxillofacial Radiology. Inclusion was based on the availability of paired pre-treatment and post-treatment digital panoramic radiographs, alongside detailed clinical records.

### Sample size calculation

The minimum required sample size was determined via sensitivity power analysis using G*Power software (G*Power software version 3.1.9.7; Heinrich Heine University, Düsseldorf, Germany). For intra-group comparisons (pre- versus post-treatment using the Wilcoxon Signed-Rank Test), an effect size of d_z_ = 0.40 (medium) was targeted. With a total sample of 101 participants, the achieved statistical power was 97.4% (α = 0.05). For inter-group comparisons (prefabricated fiber versus metal posts using the Mann–Whitney U Test), an effect size of d = 0.50 (medium) was considered. Two groups of *n* = 50 and *n* = 51 participants achieved a statistical power of 88.9% (α = 0.05). These metrics confirm that the study was adequately powered to detect clinically significant differences in FD values.

### Inclusion and exclusion criteria

Inclusion Criteria:

- Patients aged ≥ 18 years with confirmed skeletal maturity.
- Teeth restored with either prefabricated fiber or metal posts in the mandibular canine, premolar or molar region.
- Availability of standardized, high resolution digital panoramic radiographs at two distinct time points: pre-treatment and post-treatment.
- Absence of systemic conditions (e.g., uncontrolled diabetes, hyperparathyroidism) or pharmacological treatments (e.g., bisphosphonates, chronic glucocorticoids) known to modulate bone metabolism or turnover.

Exclusion Criteria:

- A history of mandibular fractures or the presence of intraosseous pathologies (e.g., cysts, tumors, or fibro-osseous lesions) in the mandible.
- Radiographs exhibiting geometric distortion, positioning errors (e.g., patient movement), or suboptimal contrast/density that would compromise the pixel-level integrity required for fractal analysis.
- Presence of active periapical lesions, root fractures, or a history of endodontic surgery on the target tooth.
- Clinical or radiographic indicators of treatment failure (e.g., post-debonding, secondary caries, or loss of marginal integrity) during the observation period.

### Radiographic imaging protocol

To eliminate inter-device variability, only panoramic radiographs obtained using a single digital imaging system (Planmeca ProOne^®^; Planmeca Oy, Helsinki, Finland) were included in the study. Radiographs for all cases were acquired using exposure parameters of 68 kVp, 7 mA, and 10 s. To ensure longitudinal consistency and minimize positioning errors, all acquisitions were performed by a single, experienced radiologic technologist. Initial image management was conducted via Planmeca Romexis^®^ software, where brightness and contrast were calibrated. For FA, images were exported from the original RAW database in uncompressed TIFF format (Tag Image File Format) to preserve the pixel level diagnostic integrity and prevent compression artifacts associated with JPEG formats.

### Image processing, standardization, and observer calibration

The panoramic radiographs were digitally transferred in TIFF format at 600 dpi resolution and 2000 × 1750 pixels dimension. To ensure rigorous comparability across the longitudinal dataset, bit-depth and grayscale histograms were verified using ImageJ software (v1.54; National Institutes of Health, Bethesda, MD, USA). All subsequent image standardization steps including verification of brightness and contrast uniformity, as well as image dimensions were also performed within ImageJ to maintain consistent parameters across the entire dataset. All image processing and analyses were conducted on a high performance workstation (Intel^®^ Core i7-8750 H CPU @ 2.20 GHz processor, 16 GB RAM, NVIDIA GeForce GTX 1050 (4 GB) and Intel^®^ UHD Graphics 630 (128 MB) graphics cards, 17.3-inch Full HD screen, Windows 11 Professional operating system). To ensure the reliability of the measurements, a calibration session was held prior to data collection. All analyses were performed by a single experienced observer (H.Y.) who was blinded to the patient groups and time points to prevent potential bias. To assess intra-observer reproducibility, a random subset of 30% of the data was re-measured by the same observer after a 15-day interval. An Intraclass Correlation Coefficient (ICC) value of > 0.95 confirmed excellent intra-observer reliability.

### Fractal analysis protocol

**Fig. 1:**
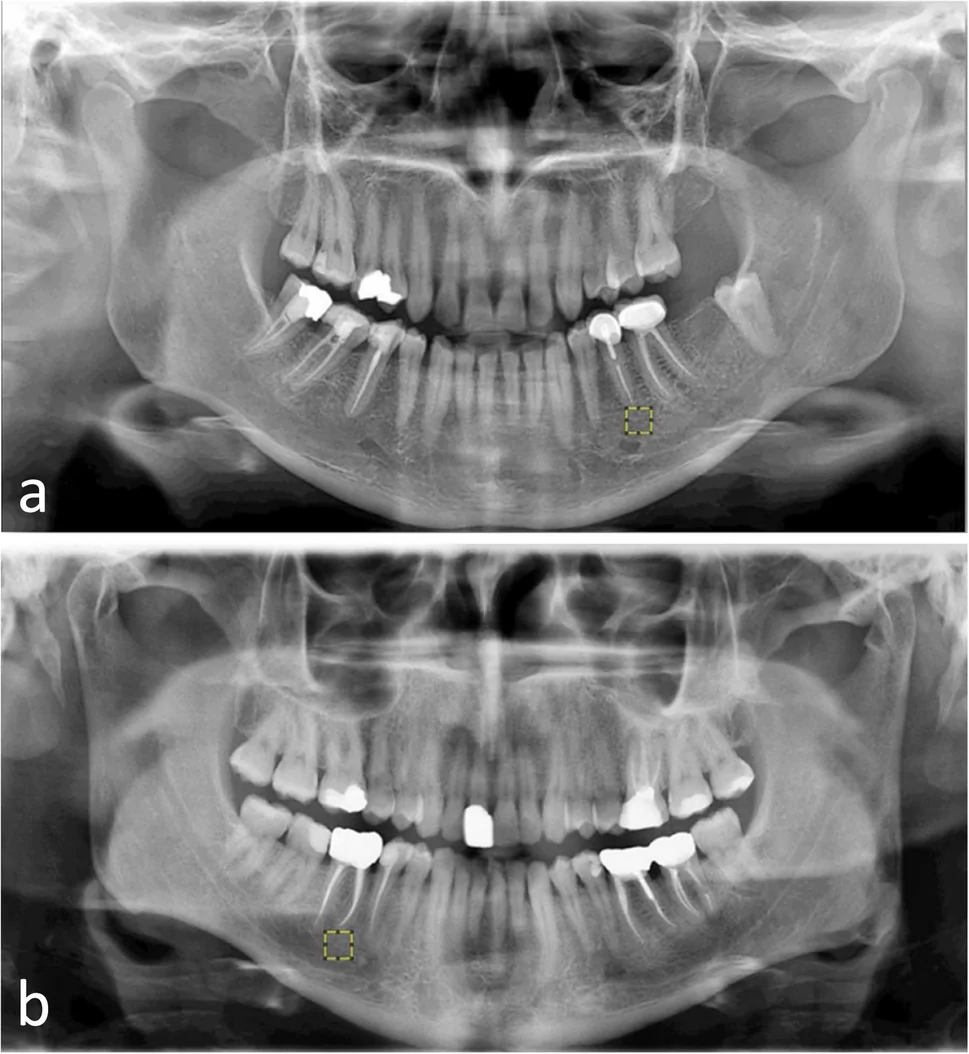
**a** Panoramic radiograph illustrating the regions of interest (ROIs) selected for fractal analysis. Representative ROI selection in the periapical region of a tooth restored with a Metal Post. **b** Representative ROI selection in the periapical region of a tooth restored with a Fiber Post

Fractal analyses were carried out using ImageJ v1.54 software (National Institutes of Health, Bethesda, MD, USA) based on the box-counting algorithm as described by White and Rudolph [19]. Regions of Interest (ROIs) were selected to represent the trabecular bone in the apical region of the mandibular canine, premolar, and molar teeth. A standardized 50 × 50 pixel ROI was determined in the peri-radicular trabecular bone of the post-restored tooth for each individual, carefully excluding anatomical structures such as the mandibular canal, mental foramen, and tooth roots (Fig. 1a and b). ROI selection intentionally avoided direct inclusion of metal post interfaces and areas showing obvious radiographic artifacts. Regions immediately adjacent to metallic structures were excluded whenever possible to minimize potential distortion during skeletonization and fractal analysis.

Measurements were obtained for each participant, encompassing both the pre-treatment and post-treatment time points, ensuring a one-year interval between measurements. The same pre-processing procedures were applied to all radiographs prior to analysis. For longitudinal standardization, the ROI on the post-treatment radiograph was manually repositioned to correspond as closely as possible to the pre-treatment ROI by using stable anatomical landmarks, including the root apex, adjacent root contours, alveolar crest morphology, and restoration margins. No automated image-registration algorithm was used. All ROI selections were performed by the same experienced observer under identical viewing conditions.

**Fig. 2:**
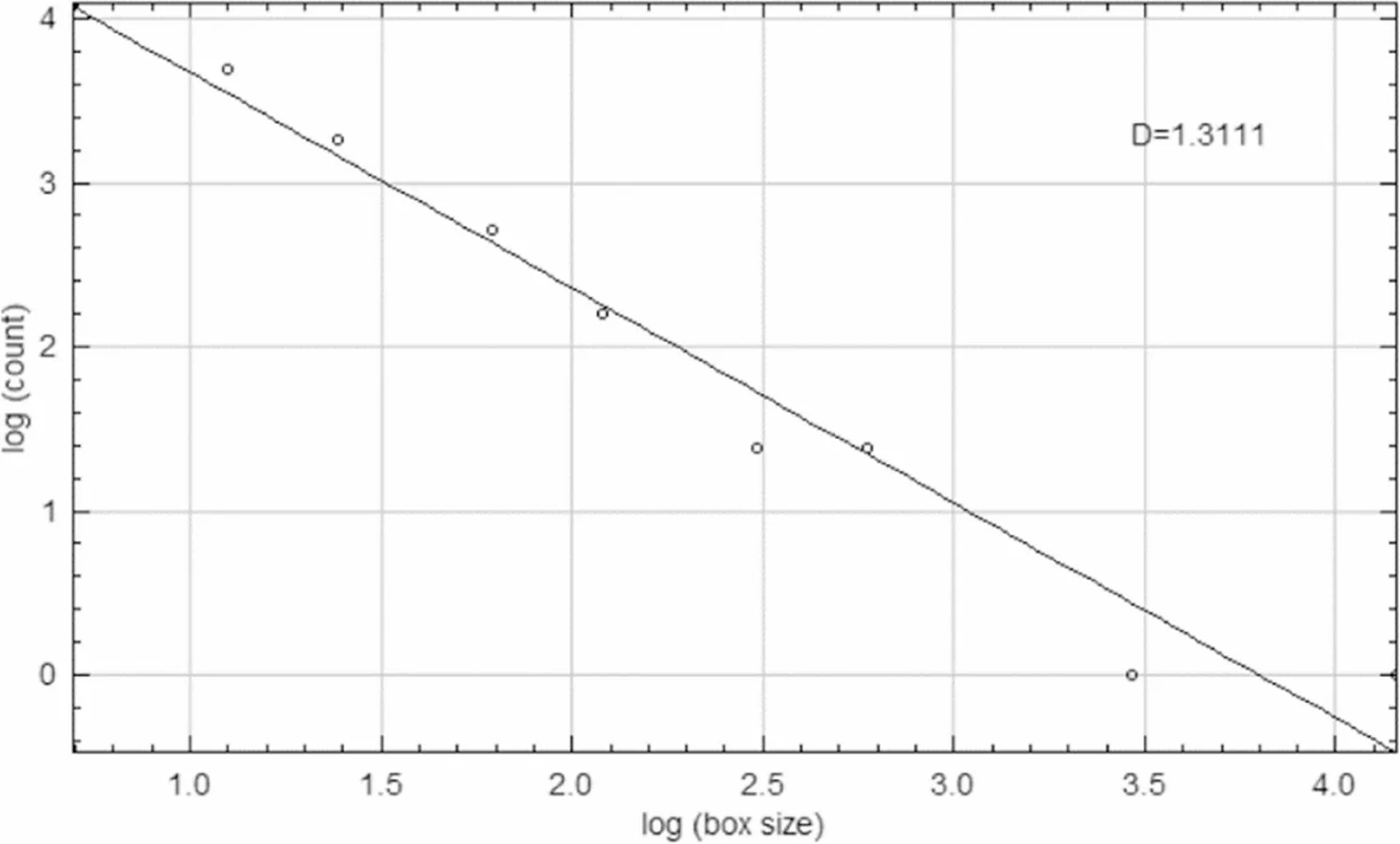
Representative log-log plot generated from the box-counting analysis, illustrating the linear regression used to derive the fractal dimension (D) value. The slope of the regression line corresponds to the fractal dimension; in this example, D = 1.3111

**Fig. 3:**
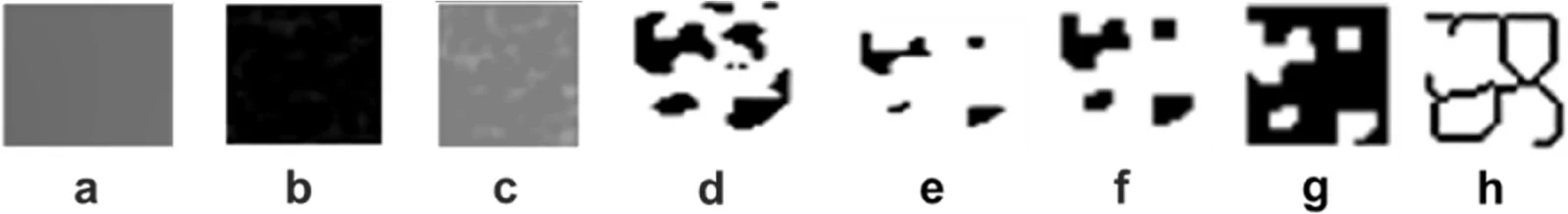
Sequential image processing steps for fractal dimension analysis. **a** Application of Gaussian filter; **b** Image subtracted from the original; **c** Grayscale adjustment; **d** Binarization; **e** Erosion; **f** Dilation; **g** Inversion; **h** Skeletonization

To standardize the digital images and eliminate background noise, a rigorous processing chain was executed using ImageJ software (v1.54, NIH). Each duplicated ROI first underwent a Gaussian blur and background subtraction, followed by the addition of a constant grayscale value of 128 to ensure intensity uniformity. The processed images were then binarized at a fixed threshold of 128, and sequential erosion and dilation were applied to refine the structural pattern. Finally, the images were skeletonized to reduce the trabecular architecture to a 1-pixel wide line, ensuring that the analysis focused on connectivity rather than trabecular thickness. The Fractal Dimension (FD) was quantified using the box-counting algorithm, where the FD value was derived as the absolute slope of the linear regression line from the log-log plot of box count (N(r)) versus box size (1/r) (Fig. 2). This quantitative metric represents the structural complexity of the mandibular trabecular bone, with higher values reflecting a more intricate microarchitecture. The sequential image processing steps for fractal dimension analysis are shown in Fig. 3.

### Statistical analysis

Statistical analyses of all data obtained in the study were performed using IBM SPSS Statistics software (version 26.0; IBM Corp., Armonk, NY, USA). The Shapiro–Wilk normality test was applied to examine the distribution characteristics of continuous variables. Nonparametric methods were preferred for comparisons since fractal analysis measurements did not show a normal distribution. The Wilcoxon signed-rank test was used to evaluate the pre- and post- treatment changes within groups, while the Mann–Whitney U test was used for comparisons between the prefabricated fiber and metal post groups. The same non-parametric test approach was followed in the evaluations based on gender. A significance level of *p* < 0.05 was accepted in all statistical evaluations.

## Results

### Demographic characteristics

A total of 101 participants were included in the final analysis, comprising 50 individuals in the prefabricated fiber post group and 51 in the prefabricated metal post group. The study population was predominantly female (72.3%, *n* = 73) compared to male (27.7%, *n* = 28), with a balanced gender distribution between the cohorts (30.0% male in the fiber group; 25.5% in the metal group). The mean age was 44.7 ± 12.8 years for the fiber group and 46.1 ± 15.2 years for the metal group, with no significant inter-group difference (t = 0.49; *p* = 0.628), confirming the baseline comparability of the study arms. The anatomical distribution of post-restored teeth across the cohorts was as follows: canines (fiber: *n* = 2; metal: *n* = 3), first premolars (fiber: *n* = 10; metal: *n* = 8), second premolars (fiber: *n* = 13; metal: *n* = 20), first molars (fiber: *n* = 15; metal: *n* = 15), and second molars (fiber: *n* = 10; metal: *n* = 5).

### Fractal dimension analysis

The Shapiro–Wilk test confirmed that FD values did not follow a normal distribution (*p* < 0.05); thus, non-parametric analysis was executed.

Intra-group Comparisons: In the fiber post group, no statistically significant difference was observed between pre-treatment (1.3416 ± 0.1129) and post-treatment (1.3477 ± 0.0934) FD values (*p* = 0.847). Similarly, the metal post group exhibited stable FD values from pre-treatment (1.3633 ± 0.0825) to post-treatment (1.3300 ± 0.1140), with no significant longitudinal changes (*p* = 0.197).

Inter-group and Subgroup Comparisons: Direct comparison between the fiber and metal post groups revealed no significant disparities at pre-treatment (*p* = 0.429) or at post-treatment (*p* = 0.671). Detailed intra-group statistical outcomes including minimum and maximum FD values are summarized in Table 1. In the classification by gender, no statistically significant difference in FD values was found between male and female participants in either group. The corresponding p-values were 0.290 (pre-treatment) and 0.597 (post-treatment) in the prefabricated fiber post group, and 0.184 (pre-treatment) and 0.239 (post-treatment) in the prefabricated metal post group, as shown in Table 2. Inter-group comparisons of FD values at pre-treatment and post-treatment time points are presented in Table 3.

**Table 1:** Descriptive statistics and intra-group comparisons of fractal dimension (FD) values for prefabricated fiber and metal post groups

Post Group	Time Point	*N*	Mean ± SD	Min	Max	Wilcoxon *p*
Fiber	Pre-treatment	50	1.3416 ± 0.1129	1.00	1.49	0.847
	Post-treatment	50	1.3477 ± 0.0934	1.11	1.48	
Metal	Pre-treatment	51	1.3633 ± 0.0825	1.19	1.48	0.197
	Post-treatment	51	1.3300 ± 0.1140	1.03	1.50	

None of the within-group comparisons reached statistical significance (all p > 0.05)

Statistical significance was set at* p *< 0.05

*SD *Standard deviation*, Min *Minimum*, Max *Maximum

**Table 2:** Fractal dimension (FD) values stratified by gender within prefabricated fiber and metal post groups

Post Group	Time Point	Gender	*N*	Mean ± SD	Mann-Whitney U *p*
Fiber	Pre-treatment	Male	15	1.3551 ± 0.1106	0.290
		Female	35	1.3358 ± 0.1150	
	Post-treatment	Male	15	1.3385 ± 0.0875	0.597
		Female	35	1.3517 ± 0.0968	
Metal	Pre-treatment	Male	13	1.3905 ± 0.0619	0.184
		Female	38	1.3540 ± 0.0872	
	Post-treatment	Male	13	1.3655 ± 0.0836	0.239
		Female	38	1.3178 ± 0.1213	

Mann-Whitney U test was used for between-gender comparisons; p < 0.05 was considered statistically significant

*SD* Standard deviation

**Table 3:** Inter-group comparisons of fractal dimension (FD) values between prefabricated fiber and metal post groups at pre-treatment and post-treatment time points

Time Point	Post Group	*N*	Mean ± SD	Min	Max	Mann-Whitney *p*
Pre-treatment	Fiber	50	1.3416 ± 0.1129	1.00	1.49	0.429
	Metal	51	1.3633 ± 0.0825	1.19	1.48	
Post-treatment	Fiber	50	1.3477 ± 0.0934	1.11	1.48	0.671
	Metal	51	1.3300 ± 0.1140	1.03	1.50	

*SD* Standard deviation, *Min* Minimum, *Max* Maximum

*p *< 0.05 was considered statistically significant

## Discussion

In this retrospective study, fractal analysis values obtained from pre- and post-treatment panoramic radiographs were compared in patients who received prefabricated fiber and prefabricated metal posts. According to the findings, no significant difference was observed between pre- and post-treatment values in either post group. Furthermore, no significant differences were found between the groups based on gender. As no statistically significant difference in fractal dimension values was detected between the fiber and metal post groups, the research hypothesis was not supported.

The fact that FD values remained stable in both post groups before and after treatment indicates that prefabricated fiber and metal posts similarly affect the microarchitecture of the surrounding trabecular bone in the short term. This finding can be interpreted to mean that mechanical stress originating from the post material is largely absorbed within the post structure or at the dentin-post interface rather than being transmitted to the bone. Indeed, according to findings from FEA studies, posts with high elastic modulus concentrate stress within the post structure, while fiber posts with low elastic modulus provide a homogeneous stress distribution in the cervical dentin; in both cases, the resultant stress transmitted to the surrounding bone tissue is thought to remain limited [5, 6, 8]. In the restoration of weakened root canal treated teeth with posts and cores, the use of materials that are more rigid than dentin causes unnatural stress distribution within the tooth. Glass fiber posts have lower elastic modulus values compared to prefabricated metal posts and distribute stress more homogeneously within the root tissue [20].

The elastic modulus of the post material being close to that of root dentin reduces stress on the root during chewing forces, thereby lowering the risk of fracture [9]. However, the relationship between elastic modulus and stress transmission to the root is not unidirectional, and the literature presents contrasting findings. For instance, FEA studies have reported that posts with low elastic modulus transfer more stress to the root, while those with high elastic modulus absorb stress and transmit less load to the root; in this view, although glass fiber posts distribute stress homogeneously, stress still concentrates in the cervical region, whereas in posts with high elastic modulus such as stainless steel, titanium, zirconia, and cast posts, stress accumulates in the cervical and apical regions along with the post itself [5, 6]. On the other hand, post materials with higher elastic moduli than dentin have also been reported to strengthen the tooth structure and reduce stress concentration in the dentin, with stress being redistributed to the interfaces and concentrated within the post structure itself [21].

Huang et al. demonstrated in their finite element analysis that root canal morphology affects force distribution depending on the material used and that stress can be directed toward the apical region. In this context, the inability to fully standardize the treated teeth in our retrospective study can be considered one of the possible reasons for not obtaining significant differences in the fractal analysis findings [7]. FEA studies have demonstrated that posts affect dentin stress under vertical load. It has been reported that materials with different elastic moduli alter stress distribution, leading to stress concentration, particularly in the cervical dentin and post system. The FEA literature indicates that these stress concentrations are related to the flexure of the tooth structure in the cervical region and to canal morphology and post characteristics in the apical region, with high stress generally concentrated at the apical end of the post and, in some studies, in the buccal third of the posts. It is also emphasized that the elastic moduli of the crown material used in the restoration can affect stress distribution independently of the post structure [21–23]. Furthermore, the presence of metallic objects in radiographs is known to produce imaging artifacts that can bias voxel-level FD values, potentially masking localized biological bone remodeling near the post [24, 25]. The type of post material does not appear to have a significant effect on FA values. This result shows that, although materials with different elastic moduli alter stress distribution within the root, they do not always produce meaningful differences in the trabecular structure evaluated by fractal analysis.

The literature indicates that excessive occlusal forces cause changes in the periodontal ligament space, lamina dura, alveolar bone, and trabecular structure, and that this is associated with differences in occlusal forces between individuals [26]. It has been reported that occlusal force differences arising during chewing affect the trabecular bone structure, leading to changes in FD values, with this effect resulting in lower FD in the dentate regions [27]. Although it has been reported that higher FD values in males are associated with a complex trabecular structure, while lower values in females are associated with a more porous structure, there are also studies that have not found a relationship between gender and FD [14, 28, 29]. Although our study found no relationship between gender and FD, it is thought that the limited sample size and imbalance in the male-female distribution may have affected the results.

FA results may be affected by the resolution, shape, location, and size of ROIs, while high resolution provides the most reliable results in FD measurements [29]. It has been suggested that ROI size may be more decisive than placement in FD calculations of alveolar bone, and that a fixed ROI position may be preferred in longitudinal studies; furthermore, it has been recommended that teeth and other anatomical structures not be included in the ROI in FD analysis [30]. In our study, analyses were performed on panoramic radiographs (in TIFF format) recorded using a standardized protocol, and the box-counting method was applied to 50 × 50 pixel ROIs representing trabecular bone. It has been argued that factors causing bone demineralization and resorption, such as periapical lesions, increase trabecular complexity by altering surface porosity, leading to an increase in FD values [14, 18]. In our study, no significant difference was observed between the pre-treatment and post-treatment FD values in either the prefabricated fiber or metal post group. This result is thought to be due to the combined effects of various factors, including the duration of the study, restoration differences, varying post lengths, and the presence of periapical lesions resulting from canal leakage. Furthermore, the one-year follow-up period might represent a stabilized clinical phase where initial bone remodeling signals, detectable by more sensitive 3D texture metrics, had already plateaued [31, 32].

Certain limitations of this study should be taken into account. The retrospective design limited the ability to fully standardize variables such as post length, diameter, and type of restoration. In addition, it is known that 2D fractal analysis applied to panoramic radiographs has more limited sensitivity compared to trabecular parameters that can be obtained with 3D CBCT. It should also be noted that metallic artifacts in radiographs may mask localized bone changes near the post. Finally, a one-year follow-up period may be insufficient to evaluate long-term bone remodeling processes.

In future prospective studies, the use of CBCT-based 3D fractal analysis with standardized post parameters and longer follow-up periods may contribute to the detection of subtle changes in bone microarchitecture. Additionally, correlating outcome measures such as clinical failure rates and tooth loss with fractal dimension values will provide a more comprehensive understanding of the long-term biomechanical effects of post systems on bone tissue.

## Conclusion

These findings indicate that the type of post material does not result in a significant difference in mandibular trabecular bone microarchitecture in the short term, as assessed by panoramic radiograph–based fractal dimension analysis. Although fiber posts retain their established advantages in esthetics, the present fractal analysis did not detect a difference in peri-radicular trabecular bone complexity between the two prefabricated systems over a one-year follow-up. This radiograph-based observation may provide clinicians with an additional consideration for material selection, particularly when esthetic, financial considerations are weighed, while broader clinical outcomes remain to be assessed in future prospective studies.

## Data Availability

The datasets used and/or analysed during the current study are available from the corresponding author on reasonable request.

## Abbreviations

CBCT: Cone beam computed tomography
FA: Fractal analysis
FD: Fractal dimension
FEA: Finite element analysis
ICC: Intraclass correlation coefficient
IRB: Institutional review board
ROI: Region of interest
SD: Standard deviation
TIFF: Tagged image file format
